# Characteristics of US Travelers to Zika Virus–Affected Countries in the Americas, March 2015–October 2016

**DOI:** 10.3201/eid2302.161292

**Published:** 2017-02

**Authors:** Sara Lammert, Allison Taylor Walker, Stefanie Erskine, Sowmya R. Rao, Douglas H. Esposito, Edward T. Ryan, Gregory K. Robbins, Regina C. LaRocque

**Affiliations:** Massachusetts General Hospital Travelers’ Advice and Immunization Center, Boston, Massachusetts, USA (S. Lammert, E.T. Ryan, G.K. Robbins, R.C. LaRocque);; Centers for Disease Control and Prevention, Atlanta, Georgia, USA (A. Taylor Walker, S. Erskine, D.H. Esposito);; Boston University Medical Center Department of Surgery, Boston (S.R. Rao);; Massachusetts General Hospital Biostatistics Center, Boston (S.R. Rao);; Harvard Medical School, Boston (E.T. Ryan, G.K. Robbins, R.C. LaRocque)

**Keywords:** Zika, viruses, international travel, Americas, infectious disease, infection

## Abstract

Zika virus has recently been introduced to the Americas and is spreading rapidly. We evaluated the characteristics of US travelers to Zika virus–affected countries who were seen at Global TravEpiNet sites during March 2015–October 2016. Nearly three quarters of travelers were men or women of reproductive age.

In 2014, a total of 30.8 million US residents traveled internationally, and 39% of trips were to the Caribbean, Central America, and South America (the Americas) (*1*). Travelers to this region are at risk for mosquitoborne illnesses, including Zika virus infection (*2*). As of November 1, 2016, a total of 49 countries and territories in the Americas have reported Zika virus transmission (*3*).

Zika virus spreads primarily through mosquito bites and sexual contact (*2*,*4*,*5*) and is of particular concern to persons of reproductive age because Zika virus infection in pregnancy can cause microcephaly and brain defects (*6*,*7*). We describe the demographics of US travelers to Zika virus–affected countries in the Americas, with a focus on persons of reproductive age.

## The Study

Global TravEpiNet (GTEN), supported by the Centers for Disease Control and Prevention (CDC), is a consortium of US clinical practices providing pretravel healthcare to international travelers. GTEN sites include academic practices, healthcare consortia, health maintenance organizations, pharmacy-based clinics, private practices, and public health clinics (*8*). We collected data on persons seen for pretravel consultation during March–October 2016 at 20 participating clinics (*8*).

We evaluated the destinations, purpose of travel, accommodations, departure month, time to departure, length of travel, and age for all travelers to Zika virus–affected countries in the Americas. We defined a man of reproductive age as being >15 years of age and a woman of reproductive age as being 15–44 years of age (*9*). Among women of reproductive age, we evaluated the frequency of pregnancy, breastfeeding, possible pregnancy in the next 3 months, and use of prescription birth control.

We considered all countries and territories with autochthonous Zika virus transmission as reported by CDC as of November 1, 2016: Anguilla, Antigua and Barbuda, Argentina, Aruba, the Bahamas, Barbados, Belize, Bolivia, Brazil, British Virgin Islands, Cayman Islands, Colombia, Costa Rica, Cuba, Dominican Republic, Ecuador, El Salvador, French Guiana, Grenada, Guadeloupe, Guatemala, Guyana, Haiti, Honduras, Jamaica, Martinique, Mexico, Netherlands Antilles, Nicaragua, Panama, Paraguay, Peru, Puerto Rico, Saint Kitts and Nevis, Saint Lucia, Saint Vincent and the Grenadines, Suriname, Trinidad and Tobago, Turks and Caicos, US Virgin Islands, and Venezuela (*3*).

A total of 22,736 travelers were seen for a pretravel consultation during March 2015–October 2016. Of these, 6,440 (28%) planned trips to >1 Zika virus–affected country in the Americas. Peru was the most common Zika virus–affected destination (accounting for 25% of all travelers to Zika virus–affected countries), followed by Brazil (12%). Of the 6,440 travelers to Zika virus–affected countries, 4,819 (75%) were persons of reproductive age.

More than half (59%) of travelers to Zika virus–affected countries were women; nearly two thirds (63%) of these women were of reproductive age (Table 1). Overall, the most common reason for travel was leisure (59%). More than one quarter (26%) of women of reproductive age were traveling for missionary work or nonmedical service work, and 15% were traveling for research or education. Only 1% of women of reproductive age were visiting friends and relatives (hereafter referred to as VFR travelers).

**Table 1:** Demographic characteristics of travelers to Zika virus–affected countries in the Americas, March 2015–October 2016*

Characteristic	All travelers, N = 6,440	Women of reproductive age, n = 2,373	Men of reproductive age, n = 2,446
Sex			
Female	3,783 (59)	2,373 (100)	0
Male	2,657 (41)	0	2,446 (100)
Median age (IQR), y	32 (22–51)	26 (20–32)	36 (25–54)
Traveler type†			
Leisure traveler	3,816 (59)	1,209 (51)	1,483 (61)
Business traveler	696 (11)	211 (9)	357 (15)
Visiting friends and relatives	110 (2)	29 (1)	30 (1)
Medical care, providing or receiving	430 (7)	242 (10)	135 (6)
Research or education	714 (11)	366 (15)	237 (10)
Mission or nonmedical service	1,288 (20)	622 (26)	439 (18)
Adventure	600 (9)	254 (11)	231 (9)
Destination type			
Urban only	1,280 (20)	456 (19)	497 (20)
Rural only	937 (15)	332 (14)	359 (15)
Both urban and rural	4,223 (66)	1,585 (67)	1,590 (65)
Accommodations†			
Camping	645 (10)	295 (12)	262 (11)
Hostel or dormitory	1,382 (21)	662 (28)	519 (21)
Home stay, relatives	362 (6)	107 (5)	110 (5)
Home stay, nonrelatives	1,021 (16)	475 (20)	366 (15)
Hotel	4,378 (68)	1,516 (64)	1,683 (69)
Cruise	427 (7)	68 (3)	177 (7)
Other	953 (15)	336 (14)	364 (15)
Country of birth, United States	5,704 (89)	2,106 (89)	2,147 (88)
Departure months			
Mar–Jun 2015	1,050 (16)	416 (18)	380 (16)
Jul–Oct 2015	1,110 (17)	389 (16)	441 (18)
Nov 2015–Feb 2016	1,278 (20)	428 (18)	490 (20)
Mar–Jun 2016	1,587 (25)	644 (27)	552 (23)
Jul–Oct 2016	1,177 (18)	423 (18)	489 (20)
After Nov 1, 2016	238 (4)	73 (3)	94 (4)
Median time to departure (IQR), d	22 (10–40)	21 (10–39)	21 (10–39)
Median length of travel (IQR), d‡	10 (7–15)	10 (7–15)	10 (7–16)
Median no. of destination countries (IQR)	1 (1–1)	1 (1–1)	1 (1–1)

*Values are no. (%) travelers except as indicated. Women of reproductive age were defined as those 15–44 y, men of reproductive age as those >15 y. †Travelers could choose >1 response. ‡Includes travel up to 2,000 d.

Less than 1% of women of reproductive age traveling to Zika virus–affected countries reported being pregnant (n = 7) or breastfeeding (n = 9) at the pretravel consultation (Table 2). Overall, 42 women (2%) reported they were planning pregnancy in the next 3 months; nearly 5% of women ages 30–39 years were planning pregnancy. Approximately one third of women (34%) reported using prescription birth control; the highest rate of prescription birth control use (44%) was among women 20–29 years of age.

**Table 2:** Reproductive status of women of reproductive age traveling to Zika virus–affected countries in the Americas, February 2015–October 2016*

Status	No. (%) travelers				
	All women, N = 2,373	Ages 15–19 y, n = 470	Ages 20–29 y, n = 1,078	Ages 30–39 y, n = 588	Ages 40–44 y, n = 237
Pregnant	7 (0.3)	0	0	6 (1.0)	1 (0.4)
Breastfeeding	9 (0.4)	0	2 (0.2)	7 (1.2)	0
Possible pregnancy in next 3 mo	42 (1.8)	0	11 (1.0)	28 (4.8)	3 (1.3)
Using prescription birth control	803 (33.8)	87 (18.6)	477 (44.3)	194 (33.0)	45 (19.0)

*Women of reproductive age were defined as those 15–44 y.

## Conclusions

Zika virus transmission has increased in the Americas. Providing pretravel counseling on mosquito bite prevention and risk for sexual transmission of Zika virus and recommending that pregnant women not travel to areas with Zika virus transmission are public health priorities. We describe the characteristics of US travelers seeking health advice before travel to Zika virus–affected countries in the Americas. Our findings suggest areas for intervention.

First, we found that three quarters of travelers seen at GTEN sites before visiting countries with Zika virus transmission in the Americas were of reproductive age. Nearly two thirds of women traveling to Zika virus–affected countries were of reproductive age, and only approximately one-third reported using prescription birth control. Five percent of women ages 30–39 years reported planning pregnancy; not all might have disclosed their plans (nearly half of all pregnancies in the United States are unplanned) (*10*). Our findings underscore that women of reproductive age, some with immediate plans for pregnancy, are traveling to Zika virus–affected countries in the Americas. CDC has issued Zika virus–related recommendations regarding pregnancy planning for travelers (http://www.cdc.gov/zika/pregnancy/thinking-about-pregnancy.html). Healthcare providers should stay abreast of these recommendations to counsel travelers to the Americas appropriately.

Second, we found that one quarter of women of reproductive age traveling to Zika virus–affected countries in the Americas were traveling for mission or nonmedical service trips; another 15% were traveling for research or education. Previous studies show that volunteer travelers are likely to pursue health information but might not adhere to mosquito avoidance measures (*11*,*12*). A survey of volunteers traveling to the Dominican Republic in 2014 demonstrated that only 30% reapplied mosquito repellant, and <5% stayed in accommodations with screens (*12*). Service organizations might consider educating on mosquito avoidance and distributing mosquito repellant and permethrin-treated clothes for appropriate destinations (*13*) when their members travel to Zika virus–affected areas. Providing bed nets should be considered for preventing other mosquitoborne diseases.

We previously reported that ≈11% of all travelers seen at GTEN sites were VFR travelers (*8**,**14*), and the US Office of Travel and Tourism Industries estimates that 27% of travelers are VFR travelers (*1*). Only 1% of the population in our study were VFR travelers. This finding suggests that VFR travelers to Zika virus–affected countries in the Americas might seek pretravel advice at a lower rate than VFR travelers to other locations (*8*) and is noteworthy because VFR travelers are at elevated risk for mosquitoborne illnesses (*2*).

Our analysis has limitations. Travelers at GTEN sites might not represent all US international travelers, and clinical practice at GTEN sites might differ from other settings where pretravel health care is provided. Also, we did not collect data on contraceptive practices in male travelers; this information would be of interest given the current recommendation for men to use condoms for >6 months after their last possible exposure to Zika virus. Last, we did not correlate dates of travel with the time that Zika virus transmission was identified in each country.

In conclusion, our findings show that many persons of reproductive age are traveling to Zika virus–affected countries in the Americas. We observed that VFR travelers represent an unexpectedly small proportion of those seeking health advice before travel to these Zika virus–affected countries; outreach efforts to increase the frequency of pretravel health encounters for these travelers are warranted. Clinicians should provide education on mosquito bite prevention for all travelers to Zika virus–affected countries and should discuss use of condoms or abstinence to reduce the risk for sexual transmission during and after travel. In addition, clinicians should assess reproductive plans, review use of effective birth control, and discuss waiting for conception when returning from areas with Zika virus.
